# Níveis Ideais de Pressão Arterial: Adeus Curva J, Bem-vindo "Quando Mais Baixo Melhor"

**DOI:** 10.36660/abc.20250150

**Published:** 2025-04-25

**Authors:** Marcio Sommer Bittencourt

**Affiliations:** 1 Universidade de Pittsburgh Departamento de Medicina Divisão de Cardiologia Pittsburgh PA EUA Divisão de Cardiologia, Departamento de Medicina, Universidade de Pittsburgh, Pittsburgh, PA – EUA

**Keywords:** Pressão Arterial, Hipertensão, Doenças Cardiovasculares

Embora a hipertensão seja um dos fatores de risco mais bem reconhecidos para doenças cardiovasculares, não há níveis claros e universais de pressão arterial definidos como ideais. Dados originais do estudo de Framingham na década de 1970 sugeriram que não havia limite de normalidade para pressão arterial alta, indicando que "quanto menor, melhor", particularmente para pressão arterial sistólica. No entanto, a reanálise subsequente dos mesmos dados sugere uma associação de curva J entre pressão arterial diastólica e resultados.^1^ Curiosamente, o padrão da curva J foi restrito à pressão arterial diastólica, uma vez que as associações da pressão arterial sistólica com a mortalidade e os resultados cardiovasculares sempre foram lineares, sem um limite claro, pelo menos para níveis tão baixos quanto 90-110^2^ mmHg. Com base em tais dados epidemiológicos, bem como no conhecimento clínico de que níveis mais baixos de pressão arterial podem causar sintomas, as diretrizes de hipertensão recomendaram uma definição parcimoniosa de meta de pressão arterial adaptada ao risco individual do paciente. Na década de 1970, a primeira versão do tratamento do *Joint National Committee* foi consistentemente indicada apenas com pressões arteriais diastólicas acima de 105 mmHg,^3^ mas desde então, as metas têm sido progressivamente menores.

Essa curva J foi reavaliada em vários estudos desde então. Uma subanálise interessante do estudo SPRINT demonstrou que, embora a pressão arterial diastólica mais baixa esteja associada a um risco aumentado de eventos cardiovasculares, uma redução ainda mais agressiva da pressão arterial sistólica para a meta do estudo ainda leva a uma redução em eventos cardiovasculares, mesmo em indivíduos com níveis mais baixos de pressão arterial diastólica.^4^ Outro grande estudo epidemiológico demonstrou que, embora a pressão arterial diastólica baixa esteja associada ao aumento de eventos, o efeito não persiste quando ajustado para idade, sexo, fatores de risco e níveis de pressão arterial sistólica.^5^ Este estudo também demonstrou que o risco aumentado associado à pressão arterial diastólica baixa foi explicado principalmente por sua associação com idade avançada e pressão arterial sistólica mais alta. Coletivamente, as evidências indicam que a PA diastólica baixa não causa resultados piores; sua associação com eventos cardiovasculares representa apenas causalidade reversa e efeitos de confusão.^6^

Com base no conhecimento de que não há aumento de risco com níveis mais baixos de pressão arterial, vários ensaios randomizados recentes avaliaram a eficácia de metas de pressão arterial mais intensas, geralmente definidas como abaixo de 130/80 mmHg. Este tópico é o foco de uma revisão sistemática no volume atual dos Arquivos Brasileiros de Cardiologia por Brandão et al.^7^ Os nove estudos incluídos na revisão demonstram consistentemente uma redução de 13 a 17% no risco relativo de eventos cardiovasculares com uma meta de pressão arterial mais rigorosa quando comparada à meta usual de 140/90 mmHg.

Em consonância com a evidência de que metas de pressão arterial mais baixas reduzem eventos e a evidência consistente documentando ausência de qualquer aumento de risco associada a níveis de pressão arterial diastólica mais baixos, este manuscrito também fornece recomendações sociais formais para usar <130/80 mmHg como a meta para o nível de pressão arterial na população adulta em geral, incluindo indivíduos mais velhos. Reconhecer a segurança e o valor de metas de pressão arterial mais baixas pela Sociedade Brasileira de Cardiologia alinha as recomendações da nossa sociedade com a literatura contemporânea de outras regiões, como as publicadas recentemente pela Sociedade Europeia de Cardiologia em suas diretrizes de hipertensão.^8^ Embora uma abordagem personalizada deva sempre incluir um equilíbrio individualizado de riscos e benefícios do tratamento, o tratamento intenso com o objetivo de níveis mais baixos de pressão arterial deve sempre ser considerado com uma meta de 130/80 mmHg ou menos, com a noção de que a pressão arterial diastólica baixa não deve impedir que se busque esses níveis, a menos que seja limitado pelos sintomas.
