# Supplementary material for: Níveis Ideais de Pressão Arterial: Adeus Curva J, Bem-vindo "Quando Mais Baixo Melhor"
Source: Arq Bras Cardiol. 2025 Apr 25;122(3):e20250150. [Article in Portuguese] doi: 10.36660/abc.20250150 (PMC12094285; doi:10.36660/abc.20250150)
Supplement: Translation

# Translation: Ideal Blood Pressure Levels: So Long the J-Curve, Lower is Better

**DOI:** 10.36660/abc.20250150i

**Authors:** Marcio Sommer Bittencourt

**Affiliations:** 1 Pittsburgh PA USA Divisão de Cardiologia, Departamento de Medicina, Universidade de Pittsburgh, Pittsburgh, PA – USA

**Keywords:** Arterial Pressure, Hypertension, Cardiovascular Diseases

While hypertension is one of the most well-recognized risk factors for cardiovascular disease, there are no clear universally accepted ideal blood pressure levels. Original data from the Framingham heart study in the 1970s suggested no normality threshold for high blood pressure, indicating that "the lower, the better", particularly for systolic blood pressure. However, subsequent reanalysis of the same data suggests a J-curve association between diastolic blood pressure and outcomes.^1^Interestingly, the J-curve pattern was restricted to the diastolic blood pressure as the associations of systolic blood pressure with mortality and cardiovascular outcomes have always been linear with no clear threshold at least to levels as low as 90-110^2^mmHg. Based on such epidemiological data as well as clinical knowledge that lower blood pressure levels may cause symptoms, hypertension guidelines have recommended a parsimonious definition of blood pressure target tailored to the individual patient risk. In the 1970s, the first version of the Joint National Committee treatment was only consistently indicated with diastolic blood pressures above 105 mmHg,^3^but since then, targets have been progressively lower.

This J-curve has been reassessed in multiple studies since then. An interesting sub-analysis of the SPRINT trial demonstrated that although lower diastolic blood pressure is associated with an increased risk of cardiovascular events, further aggressive reduction of systolic blood pressure to the study target still leads to a reduction in cardiovascular events, even in individuals with lower diastolic blood pressure levels.^4^Another large epidemiologic study demonstrated that while low diastolic blood pressure is associated with increased events, the effect is no longer present once adjusted for age, sex, risk factors, and systolic blood pressure levels.^5^This study also demonstrated that the increased risk associated with low diastolic blood pressure was mostly explained by its association with older age and higher systolic blood pressure. Collectively, the evidence indicates that low diastolic BP does not cause worse outcomes; its association with cardiovascular events only represents reverse causation and confounding effects.^6^

Building on the knowledge that there is no increase in risk with lower blood pressure levels, several recent randomized trials have evaluated the efficacy of more intense blood pressure targets, usually defined as below 130 / 80 mmHg. This topic is the focus of a systematic review in the current volume of *Arquivos Brasileiros de Cardiologia* by Brandão et al.^7^The nine studies included in the review consistently demonstrate a 13 – 17% relative risk reduction in cardiovascular events with a more stringent blood pressure target when compared to the usual target of 140/90 mmHg.

In line with the evidence that lower blood pressure targets reduce events and the consistent evidence documenting no increased risk of lower diastolic blood pressure levels, this manuscript also provides formal societal recommendations to use <130/80 mmHg as the target for blood pressure level in the general adult population, including older individuals. Recognizing the safety and value of lower blood pressure targets by the *Sociedade Brasileira de Cardiologia* brings our society's recommendations in line with contemporary literature of other cultures, such as recently published by the European Society of Cardiology in their hypertension guidelines.^8^Although a personalized approach should always include an individualized balance of risk and benefits of treatment, intense treatment with a goal of lower blood pressure levels should always be considered with a goal of 130 / 80 mmHg or lower with the notion that low diastolic blood pressure should not preclude aiming at those levels unless limited by symptoms.
